# The DEAD-box RNA helicase 27 negatively regulates the replication of porcine reproductive and respiratory syndrome virus by mediating GP2a autophagy degradation and inducing interferon-β production

**DOI:** 10.3389/fimmu.2025.1587647

**Published:** 2025-06-12

**Authors:** Binghua Chen, Yunyan Luo, Yongjie Chen, Jingxing Wang, Jiecong Yan, Zhan He, Fangfang Li, Chunhe Guo

**Affiliations:** ^1^ Guangdong Laboratory for Lingnan Modern Agriculture, State Key Laboratory for Animal Disease Control and Prevention, Key Laboratory of Zoonosis Prevention and Control of Guangdong Province, College of Veterinary Medicine, South China Agricultural University, Guangzhou, Guangdong, China; ^2^ College of Henry Fork School of Biology and Agriculture, Shaoguan University, Shaoguan, China

**Keywords:** PRRSV, GP2a, DDX27, innate immune, yeast two-hybrid screening

## Abstract

Porcine reproductive and respiratory syndrome virus (PRRSV) causes severe economic losses to the swine industry, with its replication and immune evasion mechanisms remaining incompletely understood. While DEAD-box helicases (DDXs) are known to either promote or inhibit viral infections, no prior studies have explored the role of DDX27 in viral pathogenesis. Here, we investigated the role of DDX27 in PRRSV infection. PRRSV infection induced the upregulation of endogenous DDX27 mRNA without affecting protein levels in Marc-145 cells. Functional studies revealed that overexpression of DDX27 significantly inhibited PRRSV N protein and mRNA accumulations, while silencing DDX27 enhanced viral replication. Using yeast two-hybrid and co-immunoprecipitation assays, we identified a specific interaction between DDX27 and the viral structural protein GP2a, but not with GP3, M, or non-structural proteins. Mechanistically, DDX27 promoted GP2a degradation via mediating selective autophagy pathway and activated IFN-β production, thereby suppressing PRRSV replication and enhancing host immune responses. These findings reveal DDX27 as a novel antiviral factor that targets PRRSV through dual mechanisms. This study broadens our understanding of the DDX family’s role in PRRSV infection and highlights DDX27 as a potential therapeutic target for controlling PRRSV.

## 1 Introduction

Porcine reproductive and respiratory syndrome virus (PRRSV) is a highly contagious RNA virus that has caused significant economic losses in the global swine industry. PRRSV primarily infects macrophages, leading to reproductive failure in sows and respiratory disease in piglets. Its ability to evade the host immune system exacerbates the challenge of controlling the disease, often resulting in persistent infections and secondary complications (1–4). As a member of the *Arteriviridae* family, PRRSV is characterized by a positive-sense single-stranded RNA genome encoding multiple structural and non-structural proteins that are crucial for viral replication, assembly, and immune evasion (3, 5, 6). Despite decades of research, the current vaccine strategies provide limited protection due to viral heterogeneity and immune escape mechanisms. Therefore, identifying host factors that regulate PRRSV replication and host immune responses is essential for developing effective antiviral strategies and improving disease control measures.

The DEAD-box RNA helicase family (DDX), named for the conserved Asp-Glu-Ala-Asp (DEAD) motif, comprises ATP-dependent RNA-binding proteins that play vital roles in RNA metabolism. These helicases participate in diverse processes such as RNA splicing, ribosome biogenesis, mRNA translation, RNA degradation, and stress granule assembly (7). Beyond their essential cellular roles, DDX proteins have emerged as key regulators in host-pathogen interactions, especially during viral infections (8). By sensing viral RNAs, modulating host immune signaling pathways, or interacting with viral components, DDX proteins function as either facilitators or inhibitors of viral replication (7, 9–13). Their involvement in innate immune pathways, such as the type I interferon (IFN) response, highlights their critical role in antiviral defense. However, DDX proteins also demonstrate a duality in function, as certain members promote viral replication by assisting viral RNA synthesis or translation, underscoring the functional diversity within the family (7, 14–16).

DEAD-box helicase 27 (DDX27), a member of the DEAD-box RNA helicase family, has been primarily studied for its role in ribosomal RNA (rRNA) processing and ribosome biogenesis (17). Structurally, DDX27 contains conserved helicase motifs critical for ATP hydrolysis and RNA unwinding, alongside additional domains that may mediate specific interactions with cellular or viral components (18, 19). DDX27 is predominantly localized in the nucleolus but can shuttle to the cytoplasm under certain conditions, suggesting its potential involvement in various cellular processes (20, 21). Beyond its role in rRNA maturation, DDX27 has been implicated in regulating cellular proliferation and maintaining genomic stability. Despite its importance in fundamental cellular functions, the role of DDX27 in host immunity or viral infections remains poorly understood, making it an intriguing candidate for further investigation in the context of viral pathogenesis.

Numerous studies have highlighted the dual roles of DDX family members in viral infections. Some DDX helicases, such as DEAD-box helicase 3 (DDX3) and DEAD-box helicase 58 (DDX58) (RIG-I), exhibit potent antiviral activity by activating type I interferon signaling pathways (22). For example, DDX3 enhances interferon-β (IFN-β) production through TBK1-IRF3 activation, while DDX58 (RIG-I) serves as a viral RNA sensor, initiating downstream antiviral signaling (23, 24). Similarly, DDX21 cooperates with RIG-I to amplify antiviral immune responses (25). On the other hand, certain DDX proteins have been shown to promote viral replication (11). For instance, DDX5 facilitates hepatitis C virus (HCV) RNA replication, and DDX6 supports viral RNA stability and translation for multiple RNA viruses (15). Furthermore, DDX10 inhibits PRRSV replication, but the virus counteracts its function by degrading it through SQSTM1/p62-dependent autophagy (26). Despite these insights into the roles of DDX proteins in viral infections, there is currently no evidence linking DDX27 to viral pathogenesis. Given the functional diversity of DDX helicases, understanding DDX27’s potential role in modulating viral replication or host immune responses is crucial. This study aims to uncover the antiviral functions of DDX27, particularly in the context of PRRSV infection, to broaden our understanding of host-virus interactions and identify novel therapeutic targets.

In this study, we explored the role of DDX27 in PRRSV infection and uncovered its novel antiviral functions. PRRSV infection was found to upregulate endogenous DDX27 mRNA expression in Marc-145 cells, though protein levels remained unchanged. Functional experiments revealed that overexpression of DDX27 significantly inhibited PRRSV N protein and mRNA accumulation, while silencing DDX27 had the opposite effect, thereby promoting viral replication. Using yeast two-hybrid (Y2H) screening and Co-IP assays, we identified a specific interaction between DDX27 and the PRRSV structural protein GP2a but not with other structural or non-structural proteins. Mechanistic studies demonstrated that DDX27 degraded GP2a protein, which may disrupt viral assembly, and concurrently activated IFN-β production, enhancing the host’s innate immune responses. Collectively, these findings reveal that DDX27 negatively regulates PRRSV replication through dual mechanisms: degrading GP2a and inducing IFN-β. This study provides new insights into the antiviral roles of DDX family members and highlights DDX27 as a potential therapeutic target for controlling PRRSV infection.

## 2 Materials and methods

### 2.1 Cells, plasmids, viruses and antibodies

HEK293T and Marc-145 cells were cultured in DMEM supplemented with 10% heat-inactivated fetal bovine serum (FBS) at 37 °C in a 5% CO_2_ incubator. Additionally, they were frozen using an FBS: DMEM: DMSO (5:4:1) mixture in liquid nitrogen for long-term storage. Plasmids, with pcDNA3.1 as the backbone, were fused with Myc or HA tags and used in the assays. The classical PRRSV-2 strain CH-1a was previously frozen and stored at -80 °C and used throughout the assays. The HA or Myc tag antibodies were obtained from Proteintech Biotechnology Company, while the DDX27 antibody was purchased from Sigma-Aldrich, America.

### 2.2 Plasmid construction

The full-length sequences of DDX27 and PRRSV non-structural proteins were separately amplified by PCR using specific primers listed in **Supplementary Table 1** from the cDNA of PRRSV-infected Marc-145 cells and were cloned into the pCAGGS-MCS vector with a C-terminal Myc or HA epitope tag to generate pCAGGS-DDX27-Myc and pCAGGS-Nsp(s)-HA plasmids. Positive clones were identified, sequenced, and subjected to BLAST analysis in the NCBI database.

### 2.3 TCID_50_ assay

Marc-145 cells were seeded in a 6-well plate at a density of approximately 1-2x10^6 cells per well and incubated at 37 °C in a 5% CO_2_ incubator. The CH-1a virus was serially diluted in DMEM at 10-fold intervals, and the diluted virus was added to the cells in the 6-well plate. Typically, 100 µL of each virus dilution is added to 6–10 replicate wells per dilution, followed by incubation for 4–7 days at 37°C in a CO_2_ incubator. After incubation, each well was examined under a microscope for signs of cytopathic effect (CPE), and the presence or absence of CPE was recorded for each well. Following data collection, the TCID_50_ was calculated using the Reed-Muench method with at least 3 technical replicates.

### 2.4 Quantitative RT-PCR

Total RNA was extracted from Marc-145 cells using TRizol reagent. The first strand of cDNA was synthesized via reverse transcription polymerase chain reaction (RT-PCR). Quantitative PCR (qPCR) was subsequently performed using ChamQ SYBR qPCR Master Mix (Low ROX Premixed) (Vazyme, Nanjing, China), following the manufacturer’s instructions.

### 2.5 Western blotting analysis

Cells cultured in 60-mm dishes were lysed with 200 μL of RIPA buffer (high) (Beyotime, Shanghai, China), and the mixture was incubated on ice for 30 minutes (min) before centrifugation for 5 min. The supernatant containing the proteins was transferred to a new tube and boiled for 10 min. Proteins of varying sizes were then separated using 8%-12% SDS-PAGE and transferred onto a polyvinylidene difluoride (PVDF) membrane (Millipore, Billerica, MA) at 100 V while cooling on ice for 1–2 hours (h). For western blotting, the membrane was blocked with 5% non-fat milk powder for 1 h, followed by incubation with specific primary antibodies and corresponding anti-rabbit or anti-mouse secondary antibodies. Tubulin or GAPDH antibody was used as an internal reference, detected with a mouse monoclonal antibody to ensure equal protein loading.

### 2.6 Indirect immunofluorescence assay

The Marc-145/HEK-293T cells were fixed onto microscope slides to preserve cellular structures and target antigens. Once the cell density reached 80%, they were transfected using transfection reagents. After 24 h, a specific primary antibody was applied to the samples, followed by a wash to remove any unbound primary antibody. A fluorescent-labeled secondary antibody was then added. After washing off unbound secondary antibodies, the prepared slides were visualized under a fluorescence microscope, and images were captured using a 10×objective.

### 2.7 Co-immunoprecipitation

Protein samples were extracted from cells cultured in 60-mm dishes using 250 μL of cell lysis buffer containing protease inhibitors (Beyotime, Shanghai, China). The cell lysates were incubated on ice for 30 min to ensure complete lysis. Cell disruption was enhanced by ultrasonication for 30 seconds (s). Afterwards, the lysates were centrifuged at high speed for 5 min, and the supernatant was carefully transferred to new tubes for subsequent analysis. A small aliquot of the supernatant was reserved for input detection, while the remainder was incubated with protein A/G magnetic beads (Selleck) to capture the specific antibody, in accordance with the manufacturer’s instructions. The antigen-antibody complexes were incubated for 2 h at room temperature. The beads were then washed more than five times with washing buffer (50 mM Tris, 150 mM NaCl, 0.5% detergent, pH 7.5). Subsequently, 40 μL of 1×SDS-PAGE loading buffer was added to the beads, followed by denaturation for 5 min at 100°C. The proteins were subsequently separated by SDS-PAGE for analysis.

### 2.8 Yeast two-hybrid screening assay

In the Y2H screening of the nuclear system, all nonstructural proteins in PRRSV genome were constructed into pGBKT7 vector using specific primers listed in **Supplementary Material**. Meanwhile full-length coding sequence (CDS) of DDX27 from monkeys was inset into pGADT7 vector, pGBKT7-PRRSV Nsp(s) and pGADT7-DDX27 were co-transformed into Y2H Gold cells according to the GAL4 system as described in the BD Matchmaker Library Construction and Screening Kits User Manual (Clontech, Palo Alto, CA). Interacted screening was performed on the Ade/Leu/Trp/His-deficient medium, positive control experiments were performed by co-transforming pGBK-53 and pGAD-T, while negative controls were performed with pGBK-Lam and pGAD-T.

In the Y2H screening of membrane system, according to split-ubiquitin screening theory, the subcellular localization of PRRSV structural proteins was predicted by online web: http://www.csbio.sjtu.edu.cn/bioinf/Cell-PLoc/, correspondingly, transmembrane helices in proteins were predicted by online web: https://services.healthtech.dtu.dk/services/TMHMM-2.0/. According to the predicted results, PRRSV proteins were selectively constructed into pBT3-SUC, pBT3-STE, or pBT3-N, while DDX27 was fused to pPR3-N vector. All operations were performed to refer to the DUALhunter starter kit User Manual. Interacted clones were grown onto Ade/Leu/Trp/His-deficient medium supplying 50 mM 3-Aminotriazole (3-AT), positive control experiments were performed by co-transforming pTSU2-APP and pNubG-Fe65, while negative controls were performed with pTSU2-APP and pPR3-N.

### 2.9 Dual luciferase reporter gene assay

Firefly luciferase and Renilla luciferase genes were separately inset into the expressed vector, experiment procedure was conducted according to the Dual-Luciferase Reporter Gene Assay Kit manual (Yeasen Biotechnology, Shanghai, China).

### 2.10 Gene silencing

Small interfering RNAs (siRNAs) were designed based on CDS region and synthesized by SYNBIO Technologies, Suzhou, China (**Supplementary Table S1**). Two siRNAs targeting DDX27 were designated as siDDX27-1 and siDDX27-2. These siRNAs, along with a non-targeting control siRNA (siNC), were individually transfected into Marc-145 cells using Lipofectamine 2000 (Lip2000) transfection reagent. At 48 hpt, the cells were collected for western blot analysis.

### 2.11 Statistical analysis

In this study, assays were conducted with at least two independent replications as per the instruction manual. The qPCR experiments were performed using the QuantStudio 3 Real-Time PCR System (Thermo Fisher, USA), and data were analyzed using GraphPad Prism software (version 8.0). Statistical analyses were carried out using Student’s t-test and one-way ANOVA. A difference with p-values less than 0.05 was considered statistically significant.

## 3 Results

### 3.1 PRRSV infection significantly upregulates DDX27 mRNA expression without altering its protein levels

**Figure 1:**
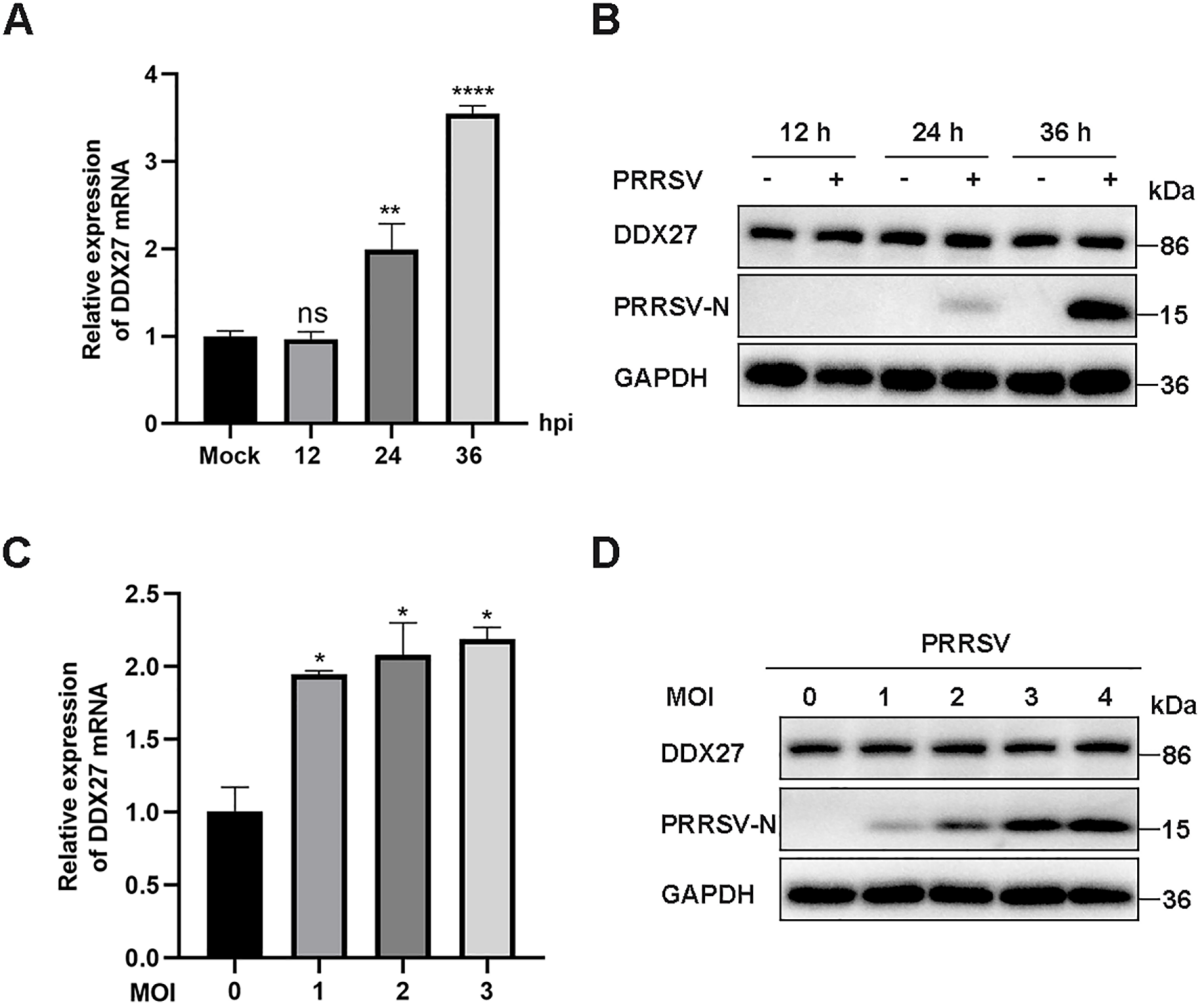
PRRSV infection induces the upregulation of endogenous DDX27 mRNA expression but has no effects on DDX27 protein levels in Marc-145 cells. **(A)** qPCR analyzed the relative expression levels of DDX27 mRNA in Marc-145 cells at 12, 24, and 36 hpi compared to uninfected cells. **(B)** Western blot detected DDX27 protein accumulations in Marc-145 cells at 12, 24, and 36 hpi and in uninfected cells. **(C)** qPCR measured DDX27 mRNA expression levels in mock-infected and PRRSV-infected cells across different multiplicities of infection (1, 2, and 3 MOI). **(D)** Western blot assessed changes in DDX27 protein expression in Marc-145 cells infected with PRRSV at various MOIs (1, 2, and 3 MOI). All experimental results were validated in at least two independent biological replicates to ensure reproducibility. *p < 0.05, **p < 0.01, ****p < 0.0001.

To investigate the changes in *Chlorocebus sabaeus* DDX27 during PRRSV infection, Marc-145 cells (a highly permissive cell line derived from the epithelial cells of a monkey kidney) were inoculated with the classical PRRSV strain CH-1a at 12, 24, or 36-hour post-infection (hpi) to determine the expression levels of DDX27. As shown in **Figure 1A**, qPCR results indicated that DDX27 mRNA levels were upregulated, increasing by approximately 3.5-fold at 36 hpi compared to non-infected cells, while DDX27 protein levels remained unchanged at 12, 24, and 36 hpi (**Figures 1A, B**). Subsequently, Marc-145 cells were infected with PRRSV at various multiplicities of infection (MOI) of 0, 1, 2, and 3 for 36 h before analyzing DDX27 mRNA expression. The qPCR results demonstrated a dose-dependent upregulation of DDX27 mRNA, with protein accumulation remaining consistent (**Figures 1C, D**). Overall, PRRSV infection leads to an upregulation of DDX27 mRNA expression but does not affect DDX27 protein levels in Marc-145 cells.

### 3.2 Ectopic expression of DDX27 restricts PRRSV replication in Marc-145 cells

**Figure 2:**
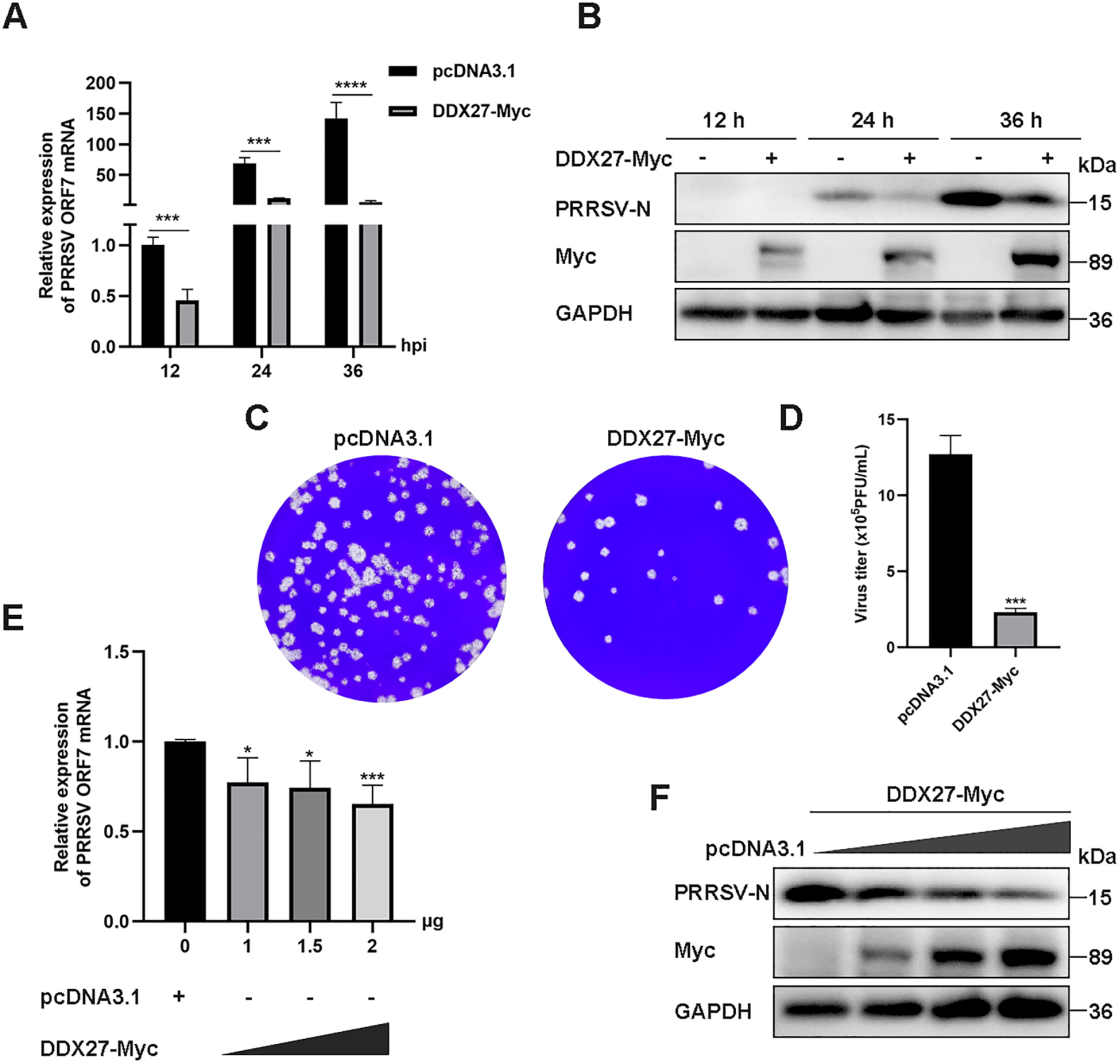
Overexpressing DDX27 inhibits PRRSV N protein and N gene mRNA accumulation, as well as virus titer in Marc-145 cells. **(A)** qPCR analyzed the relative expression levels of the N gene mRNA in Marc-145 cells infected with PRRSV for 12, 24, and 36 h, with cells overexpressing DDX27-Myc compared to those transfected with an empty vector. **(B)** Western blot assessed the accumulation of N protein in cells expressing DDX27-Myc or pcDNA3.1 during PRRSV infection for 12, 24, and 36 h, and in uninfected Marc-145 cells. **(C)** Plaque assays were conducted to analyze viral accumulation in the supernatants of Marc-145 cells infected with PRRSV for 36 h after transfection with DDX27-Myc or an empty plasmid. **(D)** Statistic analysis of three times plaque was performed by using T-test. **(E)** qPCR measured N gene mRNA expression levels in Marc-145 cells transfected with different doses of DDX27-Myc plasmids (0, 1.0, 1.5, and 2.0 μg). **(F)** Western blot detected N protein accumulation in Marc-145 cells transfected with varying doses of DDX27-Myc plasmids (0, 1.0, 1.5, and 2.0 μg). All experimental results were validated in at least two independent biological replicates to ensure reproducibility. *p < 0.05, ***p < 0.001, ****p < 0.0001.

Given that Marc-145 cells serve as a standard experimental host for PRRSV infection, we investigated whether exogenous expression of DDX27 regulates PRRSV replication within these cells. The full-length sequence of monkey DDX27 was cloned into the eukaryotic expression vector pcDNA3.1, fused with a C-terminal Myc tag. The resulting plasmid, designated DDX27-Myc, along with an empty vector (EV), was transfected into Marc-145 cells using Lipofectamine 3000 for 12 h. Subsequently, cells were infected with PRRSV and harvested at 12, 24, and 36 hpi for analysis of viral accumulation. The effect of DDX27 on viral infection was assessed by measuring PRRSV titers in culture supernatants at 36 hpi. Results showed that DDX27 mRNA levels were downregulated by approximately 0.5 to 2.5 times in the overexpressing groups compared to controls during PRRSV infection (**Figure 2A**). Western blot analysis revealed that overexpression of DDX27 significantly reduced the accumulation of PRRSV N protein compared to the control group at 24 and 36 hpi (**Figure 2B**). Besides, numbers of viral plaques and viral titer were also obviously lower in overexpressing DDX27 groups than in empty vector control groups (**Figures 2C, D**). Furthermore, a dose-dependent increase in DDX27 expression markedly decreased mRNA accumulation of the PRRSV N gene (**Figure 2E**). Additionally, reductions in PRRSV N protein accumulation were consistent with increased DDX27 expression (**Figure 2F**). Collectively, these findings confirm that overexpressing DDX27 restricts PRRSV replication in Marc-145 cells.

**Figure 3:**
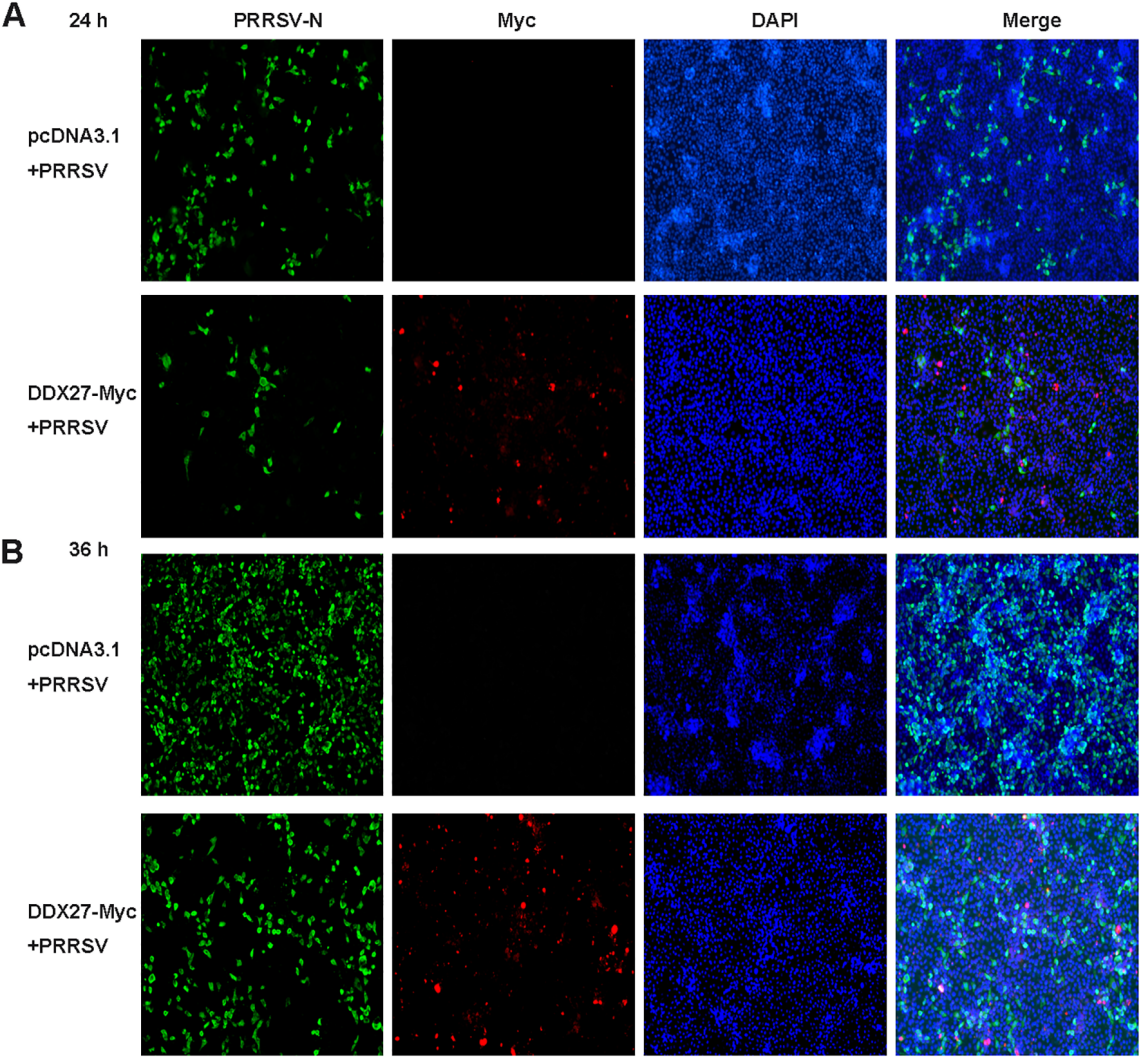
Immunofluorescence assays confirm that the expression of DDX27 reduces PRRSV infection in Marc-145 cells. **(A, B)** Transfecting DDX27-Myc or pcDNA3.1 empty vector plasmid into Marc-145 cells, at 12 hpt, PRRSV (MOI=0.5) was added to the culture medium for a continuous 12 or 24 h infection. Immunofluorescence was performed using anti-N or anti-Myc primary antibodies for hybridization. Images were captured using a fluorescence inverse microscope at 10x magnification for 24 and 36 h.

In a related immunofluorescence experiment, Marc-145 cells were transiently transfected with either DDX27-Myc or an empty vector for 12 h, followed by PRRSV infection at an MOI of 0.05 for 12 and 24 h. Immunofluorescence staining revealed a significantly reduced expression of PRRSV N protein in DDX27-Myc-transfected cells compared to the empty vector controls at both time points (**Figures 3A, B**), indicating that DDX27 overexpression effectively inhibits PRRSV replication. Myc-tagged DDX27 expression was confirmed in the cytoplasm, while robust N protein expression was observed in empty vector controls, particularly at 36 hpi. These results demonstrate that DDX27 suppresses PRRSV replication, likely through cytoplasmic interactions with viral components.

### 3.3 Knockdown of DDX27 enhances PRRSV replication in Marc-145 cells

**Figure 4:**
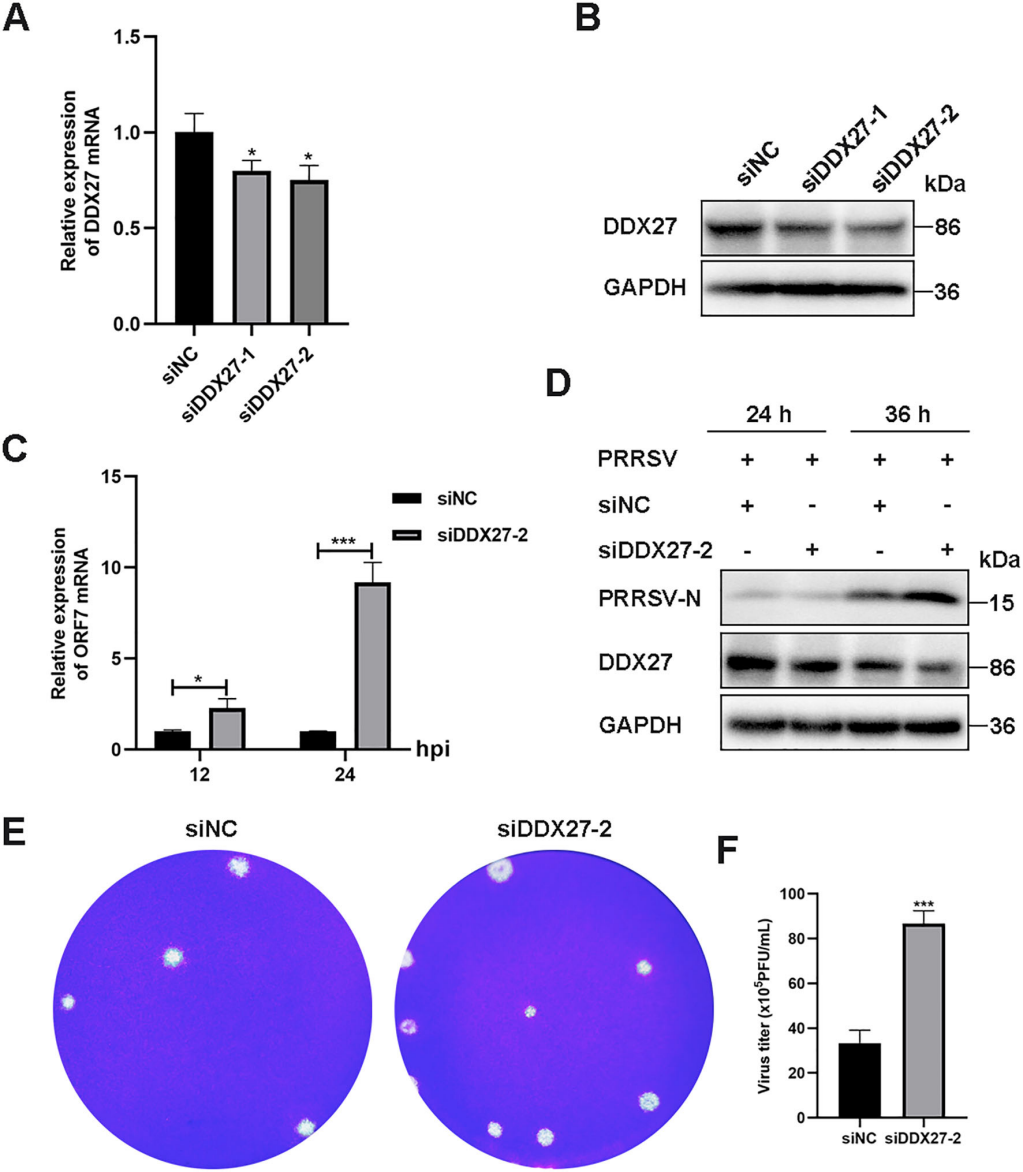
Knockdown of the endogenous DDX27 gene increases PRRSV replication in Marc-145 cells. **(A)** qPCR assessed the silencing efficiency of DDX27 in Marc-145 cells subjected to siRNA-mediated knockdown of DDX27. **(B)** Western blot evaluated the silencing efficiency of DDX27 in Marc-145 cells with siRNA-mediated downregulation of DDX27. **(C)** qPCR measured the mRNA accumulation of the N gene in DDX27-silenced and siNC (siRNA negative control) Marc-145 cells following 12 and 24 h of PRRSV infection. **(D)** Western blot analyzed the accumulation of N protein in DDX27-silenced and siNC Marc-145 cells over 24 and 36 h of PRRSV infection. **(E)** Plaque assays were conducted to analyze viral accumulation in the supernatants of Marc-145 cells infected with PRRSV for 36 h after transfection with the knockdown of DDX27 or siNC. **(F)** Statistic analysis of three times plaque was performed by using T-test in the silencing of DDX27 or control. All numbers at least two biological replicates. *p < 0.05, ***p < 0.001.

To explore the role of downregulated DDX27 in PRRSV infection, siRNA-mediated silencing targeting the coding DNA sequence (CDS) of DDX27 was performed in Marc-145 cells. Two siRNA sequences, named siDDX27–1 and siDDX27-2, were synthesized by GenePharma in Suzhou, China, and transfected into Marc-145 cells using Lipofectamine 2000. At 24 hour post-transfection (hpt), the cells were infected with PRRSV at an MOI of 0.05 for 36 h. qPCR results indicated that DDX27 expression levels were reduced to approximately 60% compared to the siRNA negative control (siNC) group (**Figure 4A**), and there was a notable reduction in DDX27 protein levels (**Figure 4B**), confirming the effective silencing of DDX27 by siRNA. Subsequently, one siRNA was selected for further experiments. After transfecting either siDDX27 or siNC into Marc-145 cells for 24 h, PRRSV was introduced at an MOI of 0.05 and the culture was maintained for 12 and 24 h. Cell samples were collected separately for RNA and protein analysis; the results showed that PRRSV N mRNA levels were significantly higher in the siDDX27 group than in the siNC group, particularly at 24 hpi, with an increase of approximately 8-fold (**Figure 4C**). Additionally, PRRSV N protein levels were significantly higher in the siDDX27 group than in the siNC group at 24 hpi (**Figure 4D**). Furthermore, the numbers of viral plaques and viral titer were also obviously higher in silencing DDX27 groups than in control groups (**Figures 4E, F**). Collectively, these findings confirm that downregulating endogenous DDX27 enhances PRRSV replication in Marc-145 cells.

### 3.4 PRRSV structural protein GP2a interacts with DDX27 as identified by Y2H screening

**Figure 5:**
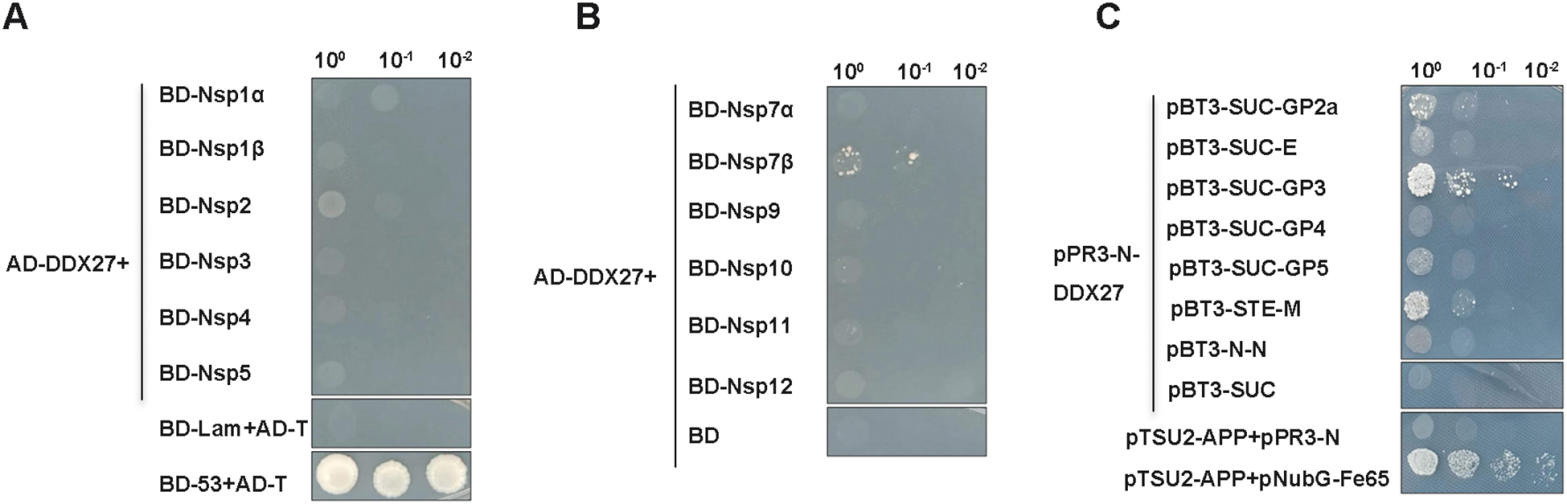
Y2H screening identifies PRRSV proteins that interact with DDX27. **(A, B)** PRRSV non-structural proteins (Nsp1α, Nsp1β, Nsp2, Nsp3, Nsp4, Nsp5, Nsp7α, Nsp7β, Nsp9, Nsp10, Nsp11, and Nsp12) were cloned into the pGBKT7 vector, while the full-length monkey DDX27 sequence was inserted into the pGADT7 vector. These constructs were co-transformed into the yeast nuclear system strain Y2H Gold cells. Positive clones were screened on auxotrophic SD/-Ade/-His/-Leu/-Trp medium, with pGBKT7-53+pGADT7-T serving as the positive control and pGADT7-DDX27+pGBDT7 and pGBKT7-Lam+pGADT7-T as negative controls. **(C)** For the structural proteins, their transmembrane helices were predicted using the online service TMHMM-2.0. These proteins were subsequently cloned into corresponding yeast membrane system vectors, resulting in the creation of bait plasmids pBT3-SUC-GP2a, pBT3-SUC-E, pBT3-SUC-GP3, pBT3-SUC-GP4, and pBT3-SUC-GP5, as well as pBT3-STE-M and pBT3-N-N. These constructs were co-transformed with pPR3-N-DDX27 into NMY151 cells, with pTSU2-AAP+pPR3-N and pPR3-N-DDX27+pBT3-SUC serving as negative controls, and pTSU2-AAP+pNubG-Fe65 as the positive control. Positive clones were screened on auxotrophic SD/-Ade/-His/-Leu/-Trp medium supplying 50 mM 3AT.

Previous studies have shown that the DDX superfamily not only positively regulates various viral replications but also inhibits PRRSV infection by interacting with viral proteins. Given this background, we investigated whether DDX27 interacts with PRRSV proteins. Using a Y2H system, which is divided into nuclear and membrane components, we constructed 12 non-structural protein-expressing plasmids (Nsp1α, Nsp1β, Nsp2, Nsp3, Nsp4, Nsp5, Nsp7α, Nsp7β, Nsp9, Nsp10, Nsp11, Nsp12) from the classical PRRSV strain CH-1a and cloned them into the yeast pGBKT7 vector. Structural proteins GP2a, E, GP3, GP4, and GP5 were sequentially cloned into the membrane system vector pBT3-SUC, while M or N sequences were inserted into yeast vectors pBT3-STE or pBT3-N, respectively. Additionally, the CDS region of DDX27 was cloned into the yeast vectors pGADT7 for the nuclear system and pPR3-N for the membrane system. In assays using the nuclear system, co-transformation of pGADT7-DDX27 with pGBKT7-(12 non-structural proteins) plasmids or a negative control pGBKT7 empty vector into Y2H Gold cells revealed minimal growth on auxotrophic SD/-Ade/-His/-Leu/-Trp medium, occurring only when AD-DDX27 was co-transformed with BD-Nsp7β (**Figures 5A, B**), indicating a weak interaction between DDX27 and Nsp7β. In the membrane system screening, co-transforming pPR3-N-DDX27 with pBT3-SUC-(GP2a, E, GP3, GP4, or GP5), or with pBT3-STE-M or pBT3-N-N into yeast NMY51 cells, and using pPR3-N-DDX27+pBT3-SUC, pTSU2-APP+pPR3-N as a negative control, and pTSU2-APP+pNubG-Fe65 as a positive control, it was found that co-transforming pPR3-N-DDX27 with pBT3-SUC-GP2a or pBT3-SUC-GP3 resulted in growth on auxotrophic SD/-Ade/-His/-Leu/-Trp medium supplemented with 50 mM 3-amino-1,2,4-Triazol (3-TA) with a few colonies comparable to the positive control (**Figure 5C**). This suggests that DDX27 interacts with GP2a, GP3 and M proteins *in vivo*.

### 3.5 Co-IP confirms the interaction between DDX27 and PRRSV structural protein GP2a

**Figure 6:**
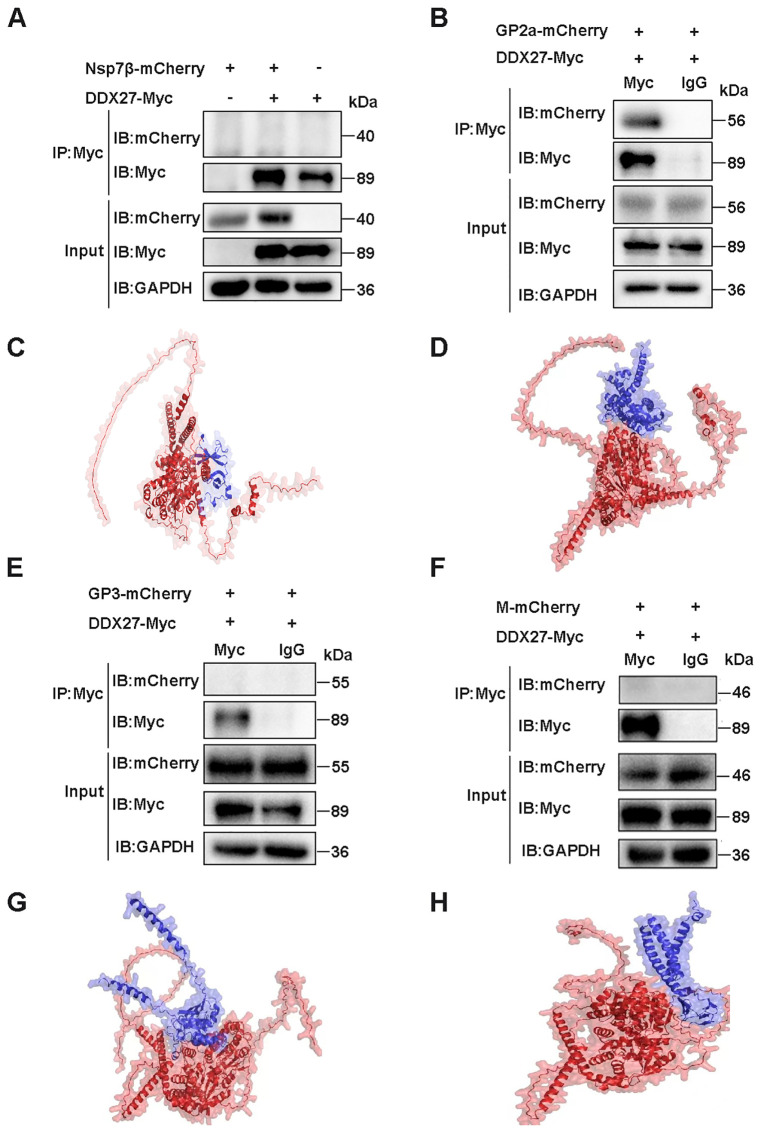
Co-IP detection of the interaction of DDX27 with Nsp7β, GP2a, GP3, or M protein and protein-protein interaction prediction by using AlphaFold3. **(A)** DDX27-Myc was co-transfected with Nsp7β-mCherry into HEK293T cells, alongside controls where each plasmid (DDX27-Myc or Nsp7β-mCherry) was transfected separately. At 24 hpt, the cells were lysed using IP lysis buffer, and anti-Myc antibodies were used to bind the beads. The immunoprecipitates were analyzed using both anti-Myc and anti-mCherry antibodies in a western blot assay. **(B)** DDX27-Myc was co-transfected with GP2a-mCherry into HEK293T cells for 24 h. The cells were then lysed using IP lysis buffer, and beads were coupled using either anti-Myc or anti-IgG antibodies. Immunoprecipitation results were analyzed via western blot using anti-Myc and anti-mCherry antibodies. **(C)** Predicted interaction structure of DDX27 binding to Nsp7β. **(D)** Predicted interaction structure of DDX27 binding to GP2a. **(E)** Co-transfection of DDX27-Myc with GP3-mCherry was performed in HEK293T cells. At 24 hpt, cells were lysed with IP lysis buffer, and the immunoprecipitation was conducted using beads coupled with either anti-Myc or anti-IgG antibodies. The precipitates were analyzed by western blot using anti-Myc and anti-mCherry antibodies. **(F)** DDX27-Myc and M-mCherry were co-transfected into HEK293T cells for 24 h. Cells were lysed using IP lysis buffer, and beads were coupled using either anti-Myc or anti-IgG antibodies. Immunoprecipitation was subsequently analyzed using anti-Myc and anti-mCherry antibodies in a western blot assay. **(G)** Predicted interaction structure of DDX27 binding to GP3. **(H)** Predicted interaction structure of DDX27 binding to M.

To validate the Y2H screening results, a co-immunoprecipitation (Co-IP) assay was employed to confirm the interaction between DDX27 and GP2a. Full-length sequences of Nsp7β, GP2a, GP3, and M proteins were individually cloned into an expression vector fused with a C-terminal mCherry tag. HEK-293T cells were co-transfected with DDX27-Myc and Nsp7β-mCherry, with single transfections of DDX27-Myc or Nsp7β-mCherry serving as controls. At 24 hpt, cells were lysed to extract total proteins for Co-IP analysis. Western blot analysis using an anti-Myc antibody for immunoprecipitation revealed that Nsp7β-mCherry was not detected in the precipitate (**Figure 6A**), indicating an absence of interaction between DDX27 and Nsp7β. Conversely, when DDX27-Myc was co-transfected with GP2a-mCherry, GP3-mCherry, or M-mCherry, and lysed with IP lysis buffer, anti-Myc antibody was coupled to beads (with anti-IgG serving as a control). The western blot results showed that only GP2a was detectable in the precipitate, while GP3 and M were not (**Figures 6B, E, F**). These findings confirm that DDX27 interacts specifically with GP2a in the Co-IP assay.

To evidence Y2H and Co-IP results, the AlphaFold3 server was utilized to predict potential interactions between monkey DDX27 and PRRSV Nsp7β, GP2a, GP3 or M proteins. The structure predicted by AlphaFold3 helps to illustrate the possible binding between DDX27 and Nsp7β, wherein the helicase core domain of DDX27 (comprising RecA1 and RecA2) maybe form a partial interface with the globular domain of Nsp7β. This binding appears to involve conserved surface residues of DDX27, potentially located within the RNA-binding groove, suggesting a weak or transient interaction. Regions highlighted in Nsp7β maybe imply minimal structural adaptation for binding (**Figure 6C**). In contrast, the predicted structure may reveal a distinct and high-affinity interaction between DDX27 and GP2a. DDX27 seems to engage GP2a via its ATPase domain, with GP2a showing conformational flexibility at its C-terminal segment, which may help to fold into the interaction surface. The predicted binding region aligns with the observed GP2a degradation, implying functional relevance in viral assembly disruption (**Figure 6D**). The predicted structure for the interaction between DDX27 and GP3 seems to show weak binding, characterized by a minimal interface area. GP3 appears to interact with peripheral regions of DDX27, situated away from the helicase core domains, implying that this interaction is less functionally significant compared to that with GP2a. Furthermore, GP3’s globular domain lacks prominent binding motifs, which may help to support the notion of a weaker interaction (**Figure 6G**). The interaction structure between DDX27 and the M protein displays minimal binding surfaces. The M protein, characterized by a compact conformation, maybe interact with DDX27’s linker regions rather than its helicase core. This predicted interaction is likely transient and may not directly contribute to DDX27’s antiviral mechanism (**Figure 6H**). Overall, these structural predictions seem to suggest that while DDX27 exhibits weak interactions with Nsp7β, GP3, and M proteins, its specific and strong interaction with GP2a likely underpins its role in PRRSV inhibition through targeted degradation and disruption of viral assembly.

### 3.6 DDX27 degrades GP2a protein via autophagy-lysosome pathway

**Figure 7:**
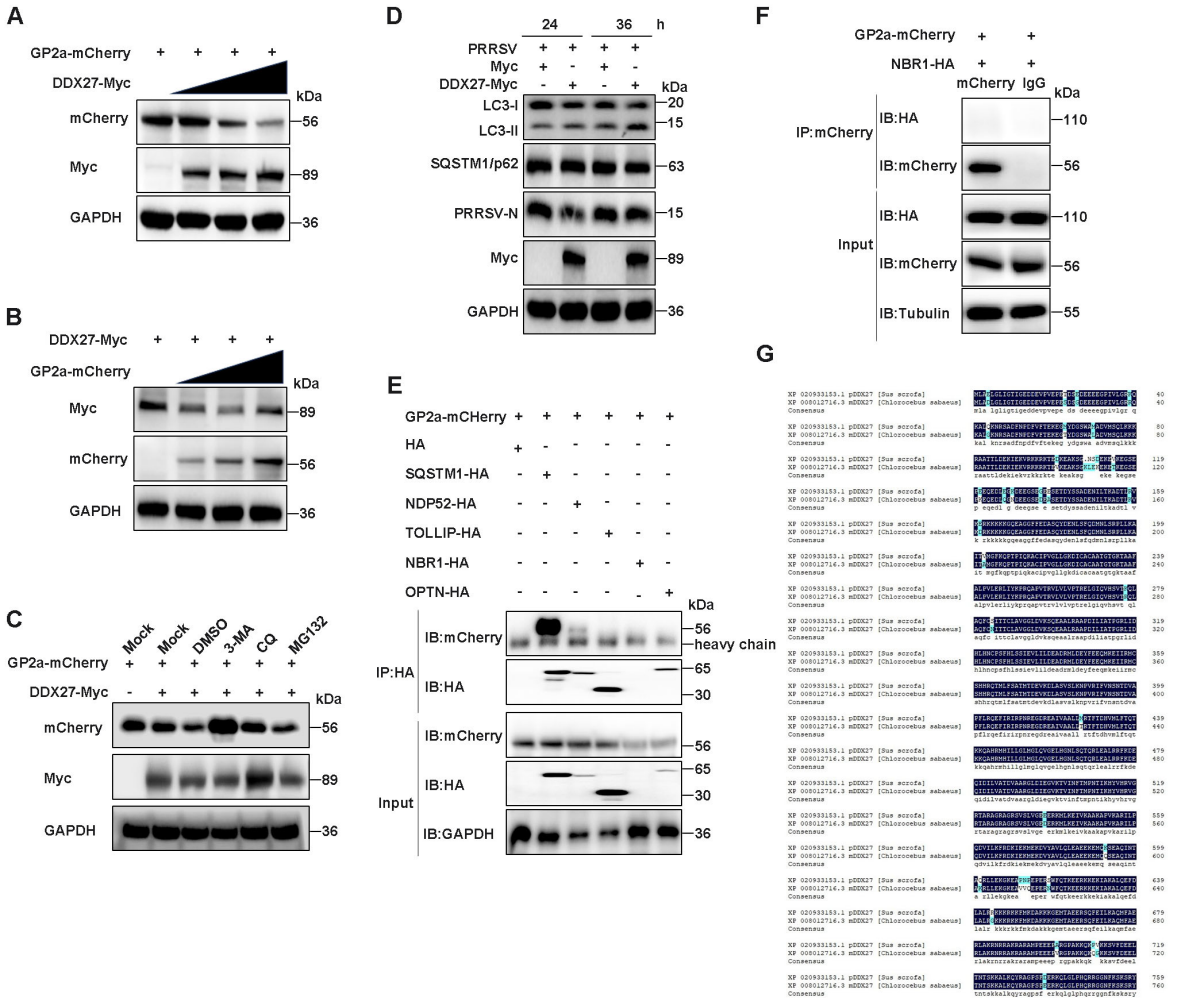
DDX27 degrades GP2a protein via the autophagy-lysosome pathway. **(A)** HEK293T cells were co-transfected with GP2a-mCherry and varying doses of DDX27-Myc plasmid for 24 h. Western blot analysis was performed to assess the protein levels of GP2a and DDX27 using specific antibodies. **(B)** HEK293T cells were co-transfected with DDX27-Myc and varying doses of GP2a-mCherry plasmid for 24 h. Western blot was used to analyze the protein levels of GP2a and DDX27 with the appropriate antibodies. **(C)** HEK293T cells were co-transfected with GP2a-mCherry and DDX27-Myc or an empty vector plasmid. At 10 hpt, cells were treated with MG132, 3-MA, and CQ inhibitors for 14 h, with DMSO as the control. Western blot analysis was used to examine the protein accumulation of GP2a. **(D)** Western blot assessed the accumulations of LC3-I/II and SQSTM1/p62 in cells expressing DDX27-Myc or pcDNA3.1 during PRRSV infection for 24 and 36 h, and in uninfected Marc-145 cells. **(E, F)** The interaction between GP2a and well-known autophagy receptors containing SQSTM1/p62, NDP52, TOLLIP, NBR1 and OPTN was detected by Co-IP. **(G)** Amino acid sequence homology from only one transcript of pig and monkeys DDX27 was performed by DNAMAN software.

To investigate the biological significance of the interaction between DDX27 and GP2a, we explored the relationship between this two proteins. Initially, DDX27-Myc was co-transfected with GP2a-mCherry into HEK293T cells in a dose-dependent manner to examine DDX27’s effect on GP2a. Western blot analysis revealed a gradual decrease in GP2a protein levels as DDX27 expression increased (**Figure 7A**), suggesting that DDX27 may promote the degradation of GP2a through their interaction. In contrast, when GP2a-mCherry was co-transfected with DDX27-Myc, the protein levels of DDX27 remained unchanged, regardless of the increasing doses of GP2a (**Figure 7B**). To determine the degradation pathway, HEK293T cells co-transfected with GP2a-mCherry and DDX27-Myc plasmids for 10 h were treated with the proteasome inhibitor MG132, the autophagy inhibitor 3-MA, or CQ, with DMSO as the control. Western blot analysis indicated that both the 3-MA and CQ-treated groups exhibited retained Nsp1β protein accumulation, with the 3-MA-treated group showing a significant rescue effect, suggesting that 3-MA and CQ, as autophagy inhibitors, inhibited Nsp1β degradation (**Figure 7C**).

To further validate DDX27’s role in mediating GP2a autophagic degradation, we performed systematic analysis of autophagy flux in PRRSV-infected Marc-145 cells. Western blot analysis revealed that DDX27 overexpression significantly increased LC3-II accumulation while slight decreasing p62 levels compared to empty vector controls (**Figure 7D**), demonstrating DDX27-dependent activation of autophagic flux during PRRSV infection. Critically, Co-IP assays identified a robust interaction between GP2a and the selective autophagy receptor SQSTM1/p62, while only a weak interaction was detected with NDP52 and no binding with other receptors (TOLLIP, NBR1 or OPTN) (**Figures 7E, F**). This suggests DDX27 likely directs GP2a for degradation through the SQSTM1/p62-mediated selective autophagy pathway. Notably, the functional relevance of these findings was supported by evolutionary conservation analysis. Sequence alignment showed 95.29% amino acid identity between porcine and African green monkey (Marc-145) DDX27 (**Figure 7G**), particularly in functional domains including the helicase core and C-terminal regions. This high conservation strongly suggests DDX27 maintains equivalent autophagy regulatory functions in PRRSV’s natural target cells (PAMs) and the experimental model system. These findings demonstrate that DDX27 promotes GP2a degradation through the autophagy-lysosome pathway.

### 3.7 DDX27 promotes selective autophagy degradation of GP2a

**Figure 8:**
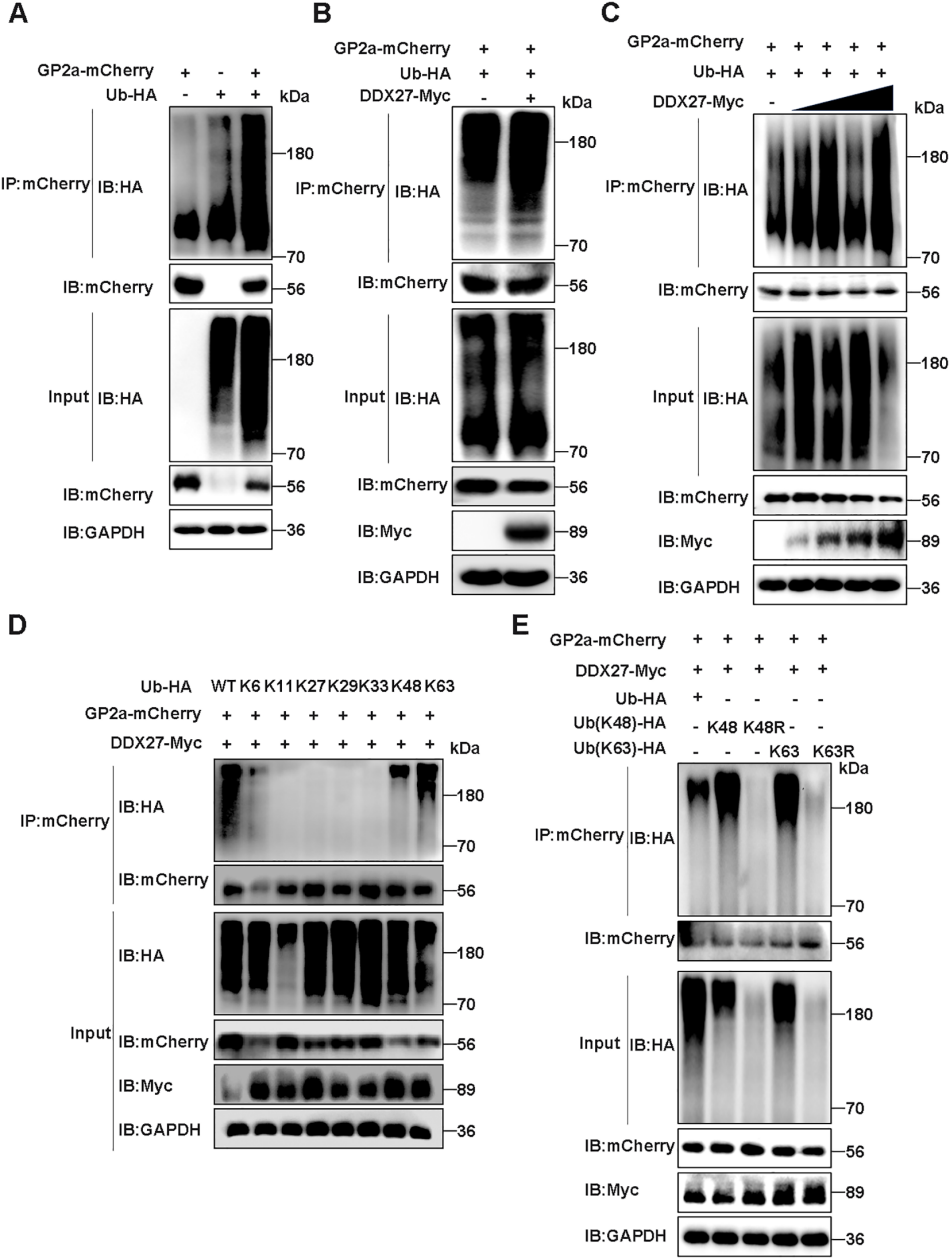
DDX27 mediates K48- and K63-linked ubiquitination of GP2a. **(A)** The ubiquitination of GP2a was analyzed in co-transfecting GP2a-mCherry and Ub-HA plasmids into HEK293T cells in Co-IP assays. **(B)** Co-transfecting GP2a-mCherrry, Ub-HA and DDX27-Myc or empty vector control into HEK293T cells, at 24 hpt, western bolt detected the ubiquitination levels of GP2a. **(C)** Co-transfecting GP2a-mCherry, Ub-HA and doze-dependent DDX27-Myc plasmids into HEK293T cells, at 24 hpt, the ubiquitination levels of GP2a were analyzed by western bolt in Co-IP assays. **(D)** Co-transfecting GP2a-mCherrry, DDX27-Myc and wild ubiquitin or ubiquitin mutants, including only reserve K6, K11, K27, K29, K33, K48 or K63 lysine mutants into HEK293T cells, at 24 hpt, the ubiquitination levels of GP2a were analyzed by Co-IP assays. **(E)** Co-transfecting GP2a-mCherrry, DDX27-Myc and wild ubiquitin or Ub mutants, including only keeping K48 or K63 mutants, and only mutating K48R or K63R mutants into HEK293T cells, at 24 hpt, the ubiquitination levels of GP2a were analyzed by western blot.

Autophagy is a universal protein degradation pathway against the host self or pathogens, in which selective autophagy recognizes specific ubiquitination target proteins. To demonstrate whether DDX27 mediates GP2a selective autophagy degradation, the ubiquitination of GP2a was studied. In Co-IP assays, GP2a-mCherry or Ub-HA plasmid was sole transfected or co-transfected into HEK293T cells, at 24 hpt, the obvious ubiquitination of GP2a was observed in co-transfection cells but not in control cells (**Figure 8A**), indicating GP2a can be modified by ubiquitination in host cells. Based on ubiquitination results, co-transfecting GP2a-mCherry, Ub-HA, DDX27-Myc plasmids or pcDNA3.1-Myc empty vector control into HEK293T cells for transient expressing 24 h, Co-IP results showed the ubiquitination levels of GP2a was obviously higher in co-transfecting DDX27-Myc groups than control groups (**Figure 8B**), illustrating the expression of DDX27 promotes GP2a ubiquitination. Besides, the ubiquitination levels of GP2a gradually enhanced companying with DDX27-Myc doze-dependent expression in Co-IP assays (**Figure 8C**), further suggesting DDX27 is an enhancer in mediating GP2a ubiquitination.

To determine the specific ubiquitin linkage types involved in DDX27-mediated GP2a degradation, we conducted systematic ubiquitination profiling in HEK293T cells co-transfected with GP2a-mCherry, DDX27-Myc, and a panel of ubiquitin mutants (K6-, K11-, K27-, K29-, K33-, K48-, or K63-only) or wild-type ubiquitin. Co-IP assays with anti-mCherry antibody followed by ubiquitin immunoblotting revealed that GP2a underwent robust polyubiquitination exclusively through K48- and K63-linked chains, as evidenced by comparable ubiquitin signals in the K48-only and K63-only groups relative to wild-type ubiquitin (**Figure 8D**). In contrast, no detectable ubiquitination was observed with other linkage-specific mutants (K6/K11/K27/K29/K33), demonstrating DDX27’s remarkable specificity for these canonical degradation signals. Next, we constructed ubiquitin mutant plasmids that only reserve K48 or K63 ubiquitin, and in reverse only mutating K48 as K48R or mutating K63 as K63R. Co-transfecting GP2a-mCherry, DDX27-Myc and wild ubiquitin or ubiquitin mutants (K48, K48R, K63, K63R) into HEK293T cells. At 24 hpt, Co-IP analyzed the ubiquitination levels of GP2a, with results showing ubiquitin mutants K48R- and K63R-linked GP2a ubiquitination were significantly inhibited than wild ubiquitin or mutants K48 or K63 (**Figure 8E**), suggesting DDX27 mediated the ubiquitination of GP2a mainly via K48- and K63-linker. Above all results, DDX27 mediated classical ubiquitination to degrade GP2a via selective autophagy.

### 3.8 DDX27 activates IFN-β luciferase activity and induces the upregulation of IFN-β expression

**Figure 9:**
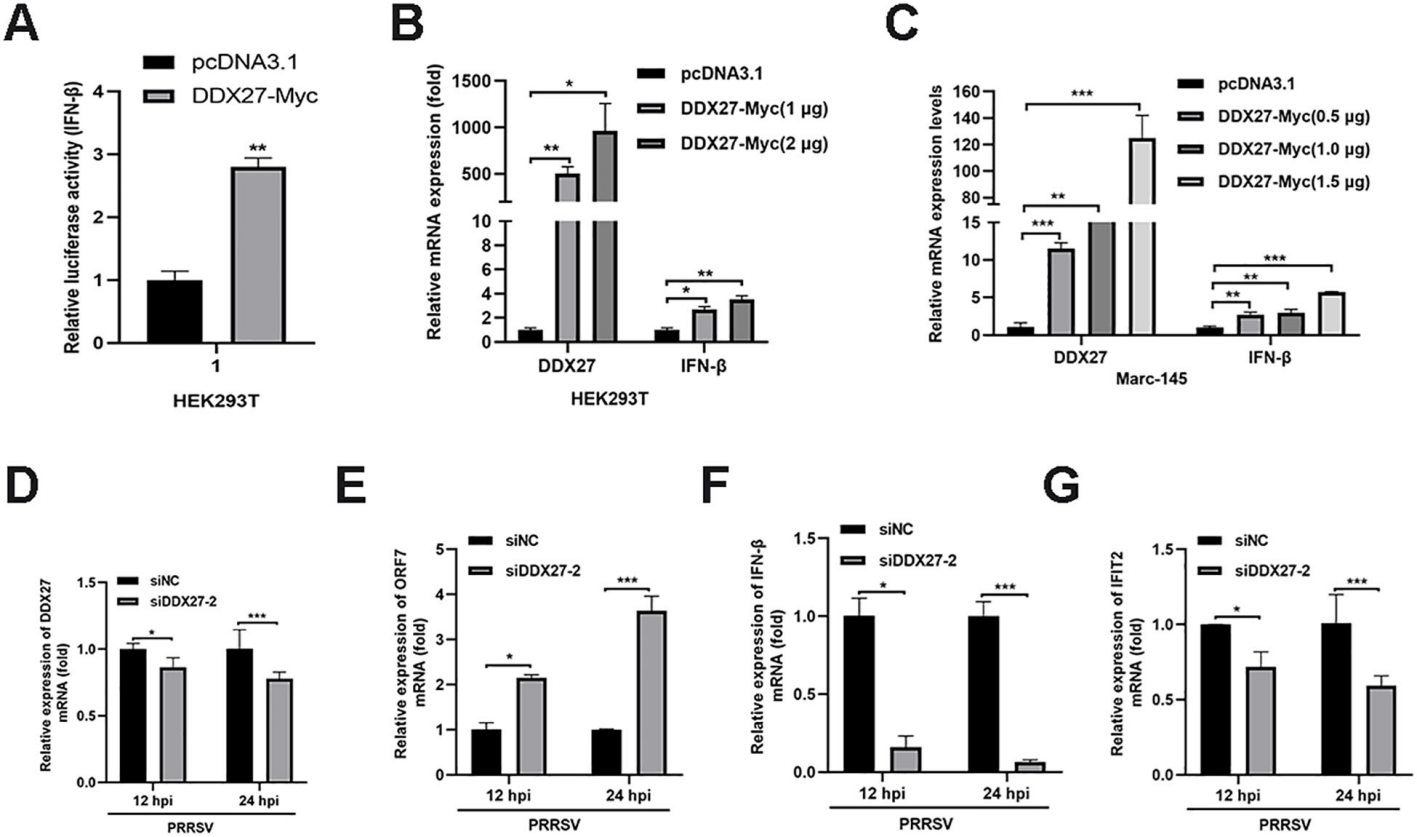
DDX27 activates IFN-β luciferase activity and induces the upregulation of IFN-β expression. **(A)** IFN-β luciferase activity was measured using the Dual-Luciferase Reporter Gene Assay Kit, following the manufacturer’s instructions. **(B)** HEK293T cells were transfected with varying doses of DDX27 or an empty vector. At 24 hpt, qPCR was performed to measure the relative expression levels of IFN-β gene. **(C)** Marc-145 cells were transfected with varying doses of DDX27 or an empty vector. At 24 hpt, qPCR was conducted to assess the relative expression levels of IFN-β. **(D-G)** Transfecting siNC or siDDX27–2 interfering RNA into Marc-145 cells, at 12 hpt, PRRSV with 0.05 MOI was used to infect these cells for 12 and 24 h. qPCR analyzed the mRNA expression levels of DDX27, ORF7, IFN-β and IFIT2 genes. All experimental results were validated in at least two independent biological replicates to ensure reproducibility. *p < 0.05, **p < 0.01, ***p < 0.001.

Previous studies have shown that DDX10 induces IFN-I production and the expression of interferon-stimulated genes (ISGs), thereby activating host innate immunity and inhibiting PRRSV replication. Based on this, we hypothesized that DDX27 may have a similar role. To test this, we cloned the IFN-β promoter into a firefly luciferase reporter plasmid. HEK293T cells were co-transfected with the IFN-β promoter Firefly luciferase plasmid, Renilla luciferase plasmid, and either DDX27-Myc or empty vector control. At 24 hpt, Dual-Luciferase activity was measured using the Dual-Luciferase Reporter Gene Assay Kit (Yeasen Biotechnology, Shanghai). Statistical analysis of triplicate data showed that DDX27 expression significantly enhanced IFN-β promoter firefly luciferase activity compared to the empty vector group (**Figure 9A**), indicating that DDX27 induces IFN-β production. Additionally, different doses of DDX27-Myc plasmids were transfected into HEK293T and Marc-145 cells for 24 h, with the empty vector as a control. qPCR analysis revealed that IFN-β mRNA expression significantly increased in a dose-dependent manner in both HEK293T and Marc-145 cells (**Figures 9B, C**). On the contrary, the silencing of DDX27 had lower mRNA expression of IFN-β and IFIT2 than the siNC control group in PRRSV-infecting Marc-145 cells for 12 h and 24 h (**Figures 9D–G**), indicating the knockdown of DDX27 inhibits PRRSV-induced innate immune IFN-β production. Collectively, these results confirm that DDX27 induces IFN-β production during PRRSV infection.

## 4 Discussion

The DEAD-box helicase family plays a critical role in the host antiviral responses and RNA metabolism, making its members important players in the regulation of viral infections (8). Numerous studies have identified key DDX helicases, including DDX3, DDX58 (RIG-I) and DDX21, as vital regulators of RNA virus infections (10, 24, 27). For example, DDX3 is widely known for its dual role in promoting IFN-β production and facilitating viral RNA translation, while DDX58 (RIG-I) serves as a sensor for viral RNA, thereby initiating robust antiviral signaling (28). DDX21 functions cooperatively with RIG-I to enhance host immune responses (29). Despite the increasing evidence regarding the roles of DDX family proteins, the function of DDX27 in viral infection, particularly in the context of PRRSV (a virus of significant economic concern in swine) has not been previously explored. In this study, we uncovered a novel role for DDX27 in suppressing PRRSV replication. Our findings demonstrated that PRRSV infection induced upregulation of endogenous DDX27 mRNA expression in Marc-145 cells, though protein levels remained unchanged, suggesting post-transcriptional or post-translational regulation. Potential mechanisms include viral-mediated translational suppression, enhanced DDX27 protein degradation, rapid protein turnover or non-productive mRNA splicing. While further studies in future work are needed to pinpoint the exact mechanism, this regulation may reflect a viral strategy to counteract DDX27’s antiviral role, with transcriptional upregulation potentially representing host compensatory responses. Functionally, DDX27 overexpression significantly inhibited PRRSV N protein and mRNA accumulation, while silencing DDX27 enhanced viral replication. Mechanistically, DDX27 specifically interacted with the structural protein GP2a, promoting its degradation and consequently inhibiting PRRSV replication. Furthermore, DDX27 was shown to activate IFN-β production, enhancing the host’s innate immune responses. These results establish DDX27 as a critical antiviral factor and highlight a novel mechanism by which DDX helicases regulate PRRSV infection.

Our findings are consistent with the established antiviral roles of DDX helicases, particularly their ability to modulate viral replication and induce host immune response (24, 30). For instance, DDX3, one of the most extensively studied members of the DDX family, has been shown to inhibit viral replication by activating the TBK1-IRF3 axis and inducing IFN-β production (23). Similarly, DDX58 (RIG-I) recognizes viral RNA and triggers downstream signaling to activate type I interferon production, leading to robust antiviral activity. In parallel, DDX27 in our study enhanced IFN-β production during PRRSV infection, suggesting that it plays a comparable role in innate immunity. However, unlike many other DDX helicases that directly recognize viral RNA, DDX27 exerts its antiviral effect by targeting a viral structural protein, GP2a, for degradation. This distinct mechanism sets DDX27 apart from other family members. While certain DDX helicases, such as DDX6, have been reported to regulate viral protein stability, DDX27 appears to exhibit a more specific interaction with PRRSV GP2a, emphasizing a unique protein-protein interaction mechanism. This specificity highlights the functional diversity of DDX helicases in targeting various stages of the viral life cycle.

Despite these alignments with the general antiviral functions of DDX helicases, our study also revealed discrepancies with previous findings, raising important questions about the unique role of DDX27 in PRRSV infection. Unlike other DDX helicases such as DDX3 and DDX58, which primarily bind viral RNA or interact with viral RNA-binding proteins (24, 31–33), our findings indicate that DDX27 does not interact with PRRSV non-structural proteins, as demonstrated by Y2H screening and Co-IP assays. This lack of interaction with non-structural proteins could be attributed to differences in the nature of viral targets, as DDX27 appears to preferentially associate with structural components like GP2a, implying DDX27 probably executes its function independent of its ATPase/helicase activity during PRRSV infection. Alternatively, the experimental methods used in our study, such as Y2H screening, may have limitations in detecting transient or weak interactions that could occur *in vivo*, is valuable for initial screening, while Co-IP data more physiologically relevant as they reflect interactions in the native cellular environment of PRRSV-infected cells. Employing other techniques, such as mass spectrometry-based interactome analysis, could yield further insights into the interactions of DDX27 with both viral and host proteins. Furthermore, variations in PRRSV strain characteristics may contribute to differences in interaction patterns and replication dynamics. PRRSV is highly heterogeneous, and genetic variations among strains could influence the ability of viral proteins to interact with host factors, including DDX27. This strain-specific variability may explain why DDX27 primarily targets GP2a in our study, while other DDX helicases, such as DDX3, have been shown to exhibit broader antiviral activity against multiple viral components.

The functional role of DDX27 in targeting GP2a also raises intriguing questions about its specificity and broader implications in viral replication. GP2a is a key structural protein involved in viral assembly and infection, and its degradation by DDX27 represents a novel antiviral strategy. The absence of interactions between DDX27 and other structural proteins, such as GP3 or M, suggests a highly selective mechanism, which could be mediated by specific domains within DDX27 or GP2a. Further studies will be needed to dissect the molecular basis of this interaction, including whether DDX27’s helicase activity is required for GP2a degradation or whether it recruits host degradation machinery, such as the ubiquitin-proteasome or autophagy pathways. Additionally, while our study demonstrates that DDX27 activates IFN-β production, the precise signaling pathway involved remains unclear. Unlike DDX3 and DDX21, which have been shown to act through the TBK1-IRF3 or RIG-I pathways (11, 25, 34), DDX27 may utilize non-canonical IFN signaling pathways. These differences underscore the functional diversity within the DDX helicase family and the potential for novel signaling mechanisms unique to DDX27.

DDX27 and DDX10 both inhibit PRRSV replication but through distinct mechanisms. DDX27 degrades the viral structural protein GP2a and activates IFN-β signaling, disrupting viral assembly and enhancing host immunity. In contrast, DDX10 primarily targets viral RNA metabolism without inducing IFN-β. The virus counters these helicases differently: DDX10 is degraded via SQSTM1/p62-mediated autophagy, while DDX27 appears to be regulated post-transcriptionally, as its protein levels remain unchanged despite mRNA upregulation (26). These differences likely stem from their functional specialization, with DDX27 acting on structural proteins in the cytoplasm and DDX10 targeting RNA processes in the nucleus. Their complementary antiviral roles underscore the diversity of helicase-mediated host defenses and PRRSV’s adaptive evasion strategies. DDX27 and DDX20 both enhance interferon pathways to inhibit viral replication, highlighting their shared role in innate immunity. However, DDX27 uniquely combines IFN-β activation with direct degradation of PRRSV’s structural protein GP2a, while DDX20 regulates NF-κB and TBK1 signaling for broader antiviral effects across multiple viruses (35, 36). These differences stem from DDX27’s PRRSV-specific strategy to counter viral immune evasion and DDX20’s specialization in transcriptional immune regulation.

In conclusion, this study provides the first evidence that DDX27 functions as an antiviral factor during PRRSV infection. By targeting the structural protein GP2a for degradation and activating IFN-β production, DDX27 exhibits a dual mechanism of antiviral action that distinguishes it from other DDX helicases. While these findings align with the general antiviral roles of DDX family members, they also highlight the unique functional attributes of DDX27, including its specific interaction with GP2a and its potential involvement in novel immune signaling pathways. Discrepancies between our results and previous studies may be explained by differences in experimental systems, viral strains, or methodological approaches, underscoring the need for further research. Future studies should focus on elucidating the molecular mechanisms underlying DDX27-mediated GP2a degradation, its precise role in IFN-β activation, and its potential functions in other RNA virus infections. By expanding our understanding of DDX27 and its antiviral mechanisms, this work lays the foundation for developing novel antiviral strategies targeting PRRSV and other RNA viruses.

## Data Availability

The raw data supporting the conclusions of this article will be made available by the authors, without undue reservation.
