# Supplementary figures and images for: The DEAD-box RNA helicase 27 negatively regulates the replication of porcine reproductive and respiratory syndrome virus by mediating GP2a autophagy degradation and inducing interferon-β production
Source: Front Immunol. 2025 Jun 12;16:1587647. doi: 10.3389/fimmu.2025.1587647 (PMC12197943; doi:10.3389/fimmu.2025.1587647)

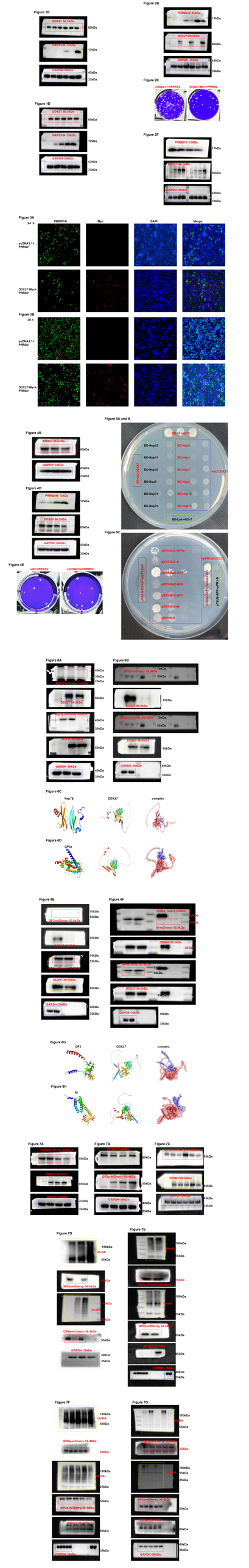

Supplement: Supplementary file 1 [file Image1.tif]
