# Supplementary material for: The DEAD-box RNA helicase 27 negatively regulates the replication of porcine reproductive and respiratory syndrome virus by mediating GP2a autophagy degradation and inducing interferon-β production
Source: Front Immunol. 2025 Jun 12;16:1587647. doi: 10.3389/fimmu.2025.1587647 (PMC12197943; doi:10.3389/fimmu.2025.1587647)
Supplement: Supplementary file 2 [file Table1.docx]

Supplemental Table S1. Primers used in this study.

| Primers | 5’ to 3’ sequence |
| --- | --- |
| mDDX27-F | CGAATTCatgcttgcggaccttggcttaatc |
| mDDX27-R | CGGATCCGcttcctcctcttgtatctggatttag |
| siDDX27-1 | GGCAGCCACUACAUUAGAUTT |
| siDDX27-2 | GACCAUGUGAUGCUGUUCATT |
| qORF7(N)-F | AAAACCAGTCCAGAGGCAAG |
| qORF7(N)-R | CGGATCAGACGCACAGTATG |
| qGAPDH-F(m) | TGACAACAGCCTCAAGATCG |
| qGAPDH-R(m) | GTCTTCTGGGTGGCAGTGAT |
| qDDX27-F(m) | gcctcagagaccgactactc |
| qDDX27-R(m) | aatcctcctgcttcctgtcc |
| Nsp1α-F | CCGGAATTCGCCACCatgtctgggatacttgatcggtgc |
| Nsp1α-R | CCGCTCGAGGcatagcacactcaaaagggcaaaagt |
| Nsp1β-F | CGAATTCatggctgacgtctatgacattggtc |
| Nsp1β-R | GCGTCGACaccgtaccatttgtgactgcca |
| Nsp2-F | atggccatggaggccgaattcATGGCTGGAAAGAGAGCAAGG |
| Nsp2-R | ccgctgcaggtcgacggatccTTAGCCCAGTAACCTGCCAAGA |
| Nsp3-F | GGAATTCGCCACCatgggcccacacctcattgctg |
| Nsp3-R | CGGATCCctcaaggagggacccgggctgag |
| Nsp4-F | CCGGAATTCGCCACCATGggcgctttcagaactcgaaagc |
| Nsp4-R | CCGCTCGAGCttccagttcgggtttggcag |
| Nsp5-F | GGAATTCGCCACCatgggaggcctttccaccgtcc |
| Nsp5-R | CGGATCCctcggcaaagtatcgcgagaagaaagcc |
| Nsp7α-F | GGAATTCGCCACCatgtcgctgactggtgccctcg |
| Nsp7α-R | CGGATCCctccagaactttcggtgggagaggg |
| Nsp7β-F | CGGGGTACCGCCACCATGaatggtcccaacgcctgg |
| Nsp7β-R | CCGCTCGAGCttcccattggactcttctattctcagg |
| Nsp9-F | agctcatcgatgcatggtaccTAGGCCACCATGGCCGCCAAGCTTTCCGTG |
| Nsp9-R | atgggtacatgctagctcgagGCTCATGATTGGACCTGAGTTTTTCC |
| Nsp10-F | GGTACCtaaGCCAGGATGgggaagaagtccagaatgtgcg |
| Nsp10-R | CCGCTCGAGCcagatctgcgcaaatagcgcg |
| Nsp11-F | CCGGAATTCGCCACCATGgaagggtcgagctccccg |
| Nsp11-R | CGCGGATCCttcaagttgaaaataggccgtcttgtc |
| Nsp12-F | CCGGAATTCGCCACCatgggccgccatttcacctgg |
| Nsp12-R | CGGATCCattcaggcctaaagttggttcaatgacaggg |
| GP2a-F | GGGTACCGCCACCatgaaatggggtctatgca |
| GP2a-R | CCCTCGAGGccgtgagttcaaaagaaaaattgcc |
| E/GP2b-F | CGGAATTCGCCACCatggggtctatgcaaagcctct |
| E/GP2b-R | CCCTCGAGCtaggatcttctgtaattgctca |
| GP3-F | agctcatcgatgcatggtaccTAAATGGCTAATAGCTGTACATTCCTCC |
| GP3-R | atgggtacatgctagctcgagTCGCCGTGCGGCACTGAG |
| GP4-F | catcattttggcaaagaattcATGGCTGCGCCCCTTCTTTTCC |
| GP4-R | atgggtacatgctagctcgagCAATTGCCAGTAGGATTGCAAAAAG |
| GP5-F | catcattttggcaaagaattcATGTTGGGGAAATACTTGACCACG |
| GP5-R | atgggtacatgctagctcgagCGAGACGACCCCATTGTTCCG |
| M/ORF6-F | CCGGAATTCGCCACCatggggtcgtctctagacgact |
| M/ORF6-R | CCGCTCGAGCtttggcatatttgacaaggtttaccact |
| N-F | CGGAATTCGCCACCatgccaaataacaacggcaagc |
| N-R | CCCTCGAGCtgctgagggtgatgctgtgacg |
| DDX27-F(m) | gtatcaacgcagagtggccattacggccATGCTTGCGGACCTTGGC |
| DDX27-R(m) | atcgaattctcgagaggccgaggcggccCTACTTCCTCCTCTTGTATC  TGGATTT |
| qIFN-β-F(m) | GCAATTGAATGGAAGGCTTGA |
| qIFN-β-R(m) | CAGCGTCCTCCTTCTGGAACT |
| qGAPDH-F(h) | tcgtggaaggactcatgacc |
| qGAPDH-F(h) | atgatgttctggagagcccc |
| qIFN-β-F(h) | tgctctcctgttgtgcttct |
| qIFN-β-R(h) | aagcctcccattcaattgcc |
| qIFIT2-F(h) | gttccattcttgccagcctc |
| qIFIT2-R(h) | cgcagatggagcagttgttt |

Notes: “m” stands for monkey species, “h” stands for human species.
